# Epigenetic regulation of complement C1Q gene expression

**DOI:** 10.3389/fimmu.2024.1498097

**Published:** 2024-12-03

**Authors:** Silvia Pegoraro, Andrea Balduit, Alessandro Mangogna, Uday Kishore, Giuseppe Ricci, Chiara Agostinis, Roberta Bulla

**Affiliations:** ^1^ Institute for Maternal and Child Health, IRCCS Burlo Garofolo, Trieste, ;Italy; ^2^ Department of Veterinary Medicine, United Arab Emirates (U.E.A.) University, Al Ain, ;United Arab Emirates; ^3^ Zayed Centre for Health Sciences, U.E.A. University, Al Ain, ;United Arab Emirates; ^4^ Department of Medical, Surgical and Health Science, University of Trieste, Trieste, ;Italy; ^5^ Department of Life Sciences, University of Trieste, Trieste, ;Italy

**Keywords:** epigenetic regulation, *C1Q*, *in silico* analysis, *C1QA*, *C1QB*, *C1QC*

## Abstract

Human C1q is a multifaceted complement protein whose functions range from activating the complement classical pathway to immunomodulation and promoting placental development and tumorigenesis. It is encoded by the *C1QA*, *C1QB*, and *C1QC* genes located on chromosome 1. C1q, unlike most complement components, has extrahepatic expression by a range of cells including macrophages, monocytes and immature dendritic cells. Its local synthesis under the conditions of inflammation and for the purpose of removal of altered self requires its strict transcriptional regulation. To delve into *C1Q* transcriptional regulation and unravel potential epigenetic influences, we conducted an *in silico* analysis utilizing a range of online tools and datasets. Co-expression analysis revealed tight coordination between *C1QA*, *C1QB*, and *C1QC* genes. Strikingly, distinct epigenetic patterns emerged across various cell types expressing or lacking these genes, with unique histone marks and DNA methylation status characterizing their regulatory landscape. Notably, the investigation extended to tumor contexts, unveiled potential epigenetic roles in malignancies. The cell type and tumor-specific histone modifications and chromatin accessibility patterns underscore the dynamic nature of epigenetic regulation of *C1Q*, providing crucial insights into the intricate mechanisms governing the expression of these immunologically significant genes. The findings provide a foundation for future investigations into targeted epigenetic modulation, offering insights into potential therapeutic avenues for immune-related disorders and cancer mediated via C1q.

## Introduction

The complement system is a tightly regulated enzymatic cascade of over 50 plasma and membrane proteins, whose activation leads to the killing and clearance of pathogens through the formation of opsonins, anaphylatoxins, and the membrane attack complex. Complement activation can occur via the classical, lectin, and alternative pathways (1, 2). The classical pathway is mainly activated by the binding of C1q to IgG- and IgM-containing immune complexes, or a range non-self (pathogen) and altered self (β-amyloid peptide, prion protein) ligands (3, 4); the lectin pathway is activated when mannan-binding lectin recognizes non-self-carbohydrate structures (5). The alternative pathway is activated when C3 spontaneously undergoes hydrolysis to generate C3b, which then interacts with various proteins, lipids, and carbohydrates on the pathogen surface (6, 7). Activation of the classical or lectin pathway leads to the breakdown of C4 and C2, resulting in the formation of C3 convertase (C4b2b), which cleaves C3 to produce C3b. In the alternative pathway, hydrolysis of the internal thioester bond in C3 produces C3(H_2_O), which binds to factor B, leading to its cleavage by factor D into Bb. This generates C3(H_2_O) Bb.

C1q is the target recognition subcomponent of the classical pathway and serves as a fundamental bridge between innate and adaptive immunity. Human C1q is a structurally complex hexameric glycoprotein (460 kDa), which associates with the Ca^2+^-dependent C1r_2_–C1s_2_ tetramer to form the pentameric C1, the first component of the complement classical pathway (8, 9). The basic subunit of C1q is composed of an N-terminal collagen-like region and a C-terminal globular domain (gC1q). The gC1q is the ligand recognition domain and has a heterotrimeric structure composed of the C-terminal halves of the A (ghA), B (ghB), and C (ghC) chains (10). Being a charge pattern recognition molecule, C1q can bind a diverse range of self and non-self ligands via its gC1q domain and then trigger the classical pathway following binding to antigen-antibody complex (11). In addition, C1q also binds to membrane blebs of apoptotic cells, thus promoting efferocytosis. Impaired clearance of apoptotic cells occurring due to C1q deficiencies can prompt autoantibody production, as in systemic lupus erythematosus (12, 13).

The *C1Q* gene cluster is located on chromosome 1 within a genomic region of ∼25 kb; it consists of three individual genes (*i.e.*, *C1QA*, *C1QC*, and *C1QB*) in the same strand orientation (+ strand), being around 4.6, 8.9, and 5.0 kb long, respectively. Each gene is composed of three exons, two of which contain the sequence that is translated into protein. Phylogenetic analysis uncovered that *C1QA*, *C1QB*, and *C1QC* may derive from duplication of a single copy of a potential ancestor identified as *C1QB;* moreover, the C1q family seems closely related to the EMILIN family (11, 14, 15). The human C1q domain containing (C1qDC) proteins are a large group of proteins, whose members have been mainly categorized into two sub-families based on their sequence homology: the larger sub-family includes the C1q-like and cerebellin-like subgroups, while the smaller is composed of EMILINs and multimerins (11, 16). Interestingly, 32 open reading frames encoding C1qDC proteins have been identified within the human genome (17). C1qDC proteins are pivotal signaling molecules that control inflammation, adaptive immunity, and energy balance, suggesting their potential exploration as therapeutics or targets in drug development (17).

Although the production of complement proteins is mostly in the liver, C1q is extrahepatically synthesized by tissue macrophages, immature dendritic cells (DCs), and other myeloid-derived cells (18–20). The evolutionary advantages and transcriptional mechanisms underlying C1q production by these potent phagocytes and antigen-presenting cells are still poorly understood. The extrahepatic synthesis of C1q is also associated with its complement-activation independent functions. Alternative roles of C1q concern its capability to promote angiogenesis and placental development (21, 22). It has been reported that C1q may act as a proangiogenic factor in several contexts, such as wound healing (23), placentation (24), and tumor progression (25), in addition to neural development, ageing, and autoimmunity (26).

Studies investigating *C1Q* transcriptional regulation have identified specific transcription factors capable of activating *C1Q* promoters. Interestingly, the transcription of the three genes is synchronized, with a 53-bp element as a regulatory region on *C1QB* promoter and two transcription factors bound to this region, namely PU.1 and IRF8 (27). PU-1 was also demonstrated to regulate decidual *C1Q* expression in pregnancy (28). In addition, the transcription factor, MafB, specifically expressed by monocytes and macrophages, binds to and activates the transcription of each *C1Q* gene (29). However, little is known regarding the mechanisms of modulation of *C1Q* expression, in particular, at the level of the tumor microenvironment (25, 30, 31).

Over the past decade, multi-omic technologies have undergone significant advancements enabling comprehensive characterization of gene expression profiles in various cells and tissues, physio-pathological conditions. These methodologies have been instrumental in elucidating intricate regulatory mechanisms governing gene expression, including epigenetic variations and protein expression dynamics. Epigenetics plays a pivotal role in establishing and maintaining cellular identity, responding dynamically to developmental cues and environmental stimuli, thereby orchestrating precise gene expression patterns that are unique to distinct cellular states (32, 33). Broadly categorized, epigenetic modifications encompass post-transcriptional histone alterations, DNA methylation, and regulatory non-coding RNA molecules. The regulatory role of histone acetylation, exemplified by H3K27ac (acetylation of lysine 27 on histone H3 protein subunit), is closely linked to transcriptional activation, whereas histone methylation exhibits a nuanced impact, capable of both activating (*e.g.*, H3K4me1 and H3K4me3) and repressing (*e.g.*, H3K27me3) transcription, depending on the specific histone site and the extent of methylation (34). Additionally, DNA methylation predominantly occurs at CpG dinucleotides within gene promoters, exerting transcriptional repression by recruiting repressive complexes to methylated promoters (35).

In the current study, we aimed to examine the transcriptional regulation of *C1QA*, *C1QB*, and *C1QC*, by exploiting publicly available tools and datasets. We particularly focused on the epigenetic dynamics underlying *C1Q* expression, and providing critical insights into the intriguing contribution of *C1Q* in the tumor microenvironment.

## Methods

### Zenbu gene expression analysis and FANTOM-CAT analysis

Human *C1Q* gene locus (comprising *C1QA*, *C1QB*, and *C1QC*) analysis was performed using Zenbu browser genomic data visualization tool from FANTOM5 project (https://fantom.gsc.riken.jp/zenbu/gLyphs/#config=FANTOM5_promoterome_hg38;loc=hg38). FANTOM5 project is established by FANTOM, an international research consortium, a part of the RIKEN research institute in Japan. Zenbu browser was used to visualize *C1QA*, *C1QB*, and *C1QC* genes, manually selecting specific tracks to show: chromosomal location, transcripts identified by Gencode, UCSC, and FANTOM6 CAT projects, transcription start site (TSS) usage and their expression in primary cells, cell lines, and tissues of the dataset (ALL libraries), retrieved by FANTOM5 CAGE project. A specific search was carried out by selecting the terms “monocyte and macrophage” and “endothelial progenitor cells”. FANTOM Cage-Associated Transcriptome (FANTOM-CAT) gene section was used to achieve sample ontology association and dynamic expression (https://fantom.gsc.riken.jp/cat/v1/#/genes). Using the FANTOM-CAT gene section browser, we retrieved the above individual information for *C1QA*, *C1QB*, and *C1QC* genes; then we compared the data for each gene with others to find similarities or differences.

### Epigenome *in silico* analysis

Zenbu genome browser epigenome view from the FANTOM5 project (https://fantom.gsc.riken.jp/zenbu/gLyphs/index.html#config=znpe_pvEJZfn_jkf_a18rB;loc=hg19), and Ensembl genome browser (https://www.ensembl.org/index.html) were used to analyze the epigenomic state of *C1Q* gene *locus*, selecting data from specific histone modification. For both analyses, we first selected the *C1Q* gene *locus*, then searched for specific cell types of interest and sorted them out. Thus, we retrieved specific epigenomic data for these cell types, selecting the following markers: DNAse I and the histone modifications (*i.e.*, H3K27ac, H3K4me3, H3K4me1, and H3K27me3 for Zenbu analysis; H3K27ac, H2K27me3, H3K4me1, H3K4me3, and H3K9me3 for Ensembl analysis).

For DNA methylation analysis on *C1Q* gene *locus* in normal cells and tissues, we used the UCSC genome browser (https://genome.ucsc.edu/) selecting the DNA methylation data from MethBase, a publicly available database as a track hub in the UCSC Genome Browser (https://smithlabresearch.org/software/methbase/). MethBase is a reference methylome database created from public Bisulfite sequencing datasets. Then, we retrieved methylome information about the cell types of our interest present in the dataset, using data from the methylation levels at individual sites and hypo- or hyper-methylated regions already identified by MethBase.

### Epigenome and gene expression analysis in tumor samples

The Shiny Methylation Analysis Resource Tool (SMART; http://www.bioinfo-zs.com/smartapp/) (36) was used for the differential methylation analysis of *C1QA*, *C1QB*, and *C1QC* genes among different tumor types and corresponding normal tissues. The results are presented as aggregation box plots showing the mean of the beta-value of methylation of all the selected probes for each gene analyzed.

The correlation between methylation levels, expressed in beta-value, and gene expression of mRNA levels (Log_2_-scaled, TPM+1) was analyzed by Spearman correlation for any given set of The Cancer Genome Atlas (TCGA) through the SMART correlation function.

For correlation analysis between *C1QA* expression levels and the expression of gene signature composed by *C1QB* and *C1QC*, we used Gene Expression Profiling Interactive Analysis 2 (GEPIA2) database (http://gepia2.cancer-pku.cn/#index) (37) using TCGA normal samples and GTEx datasets. Spearman correlation coefficient was employed. The analysis of the expression profile of each *C1Q* gene in tumor *vs* normal samples was performed by GEPIA2 Expression Analysis tool. Kidney Renal Clear Cell Carcinoma (KIRC) tumor type (*n* = 523) was selected, and matched TCGA normal and GTEx data were used as normal samples (*n* = 100). The expression values were expressed in logTPM+1 (cut off=1), *p-value* (cut off=0.01).

## Results

### Integrated analysis of TSS usage and dynamic expression patterns reveals co-expression and functional association of human *C1QA*, *C1QB*, and *C1QC* genes

**Figure 1:**
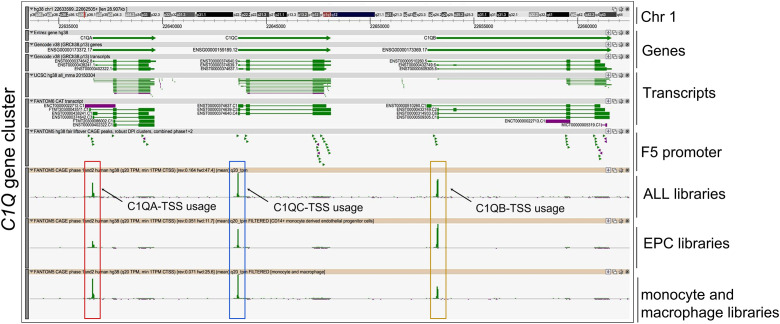
Fantom CAT analysis of *C1Q* gene cluster. Zenbu genome browser view of gene cluster for human *C1Q*. Genes and transcripts are color-coded according to their orientation in the genome (+ strand, green; – strand, purple). Upper panel reports from top to bottom: Genomic coordinates, Gencode Gene bodies and transcripts, annotated UCSC transcripts, and Robust FANTOM6-CAT transcripts, with exon (thick lines) and intron (thin lines) boundaries. FANTOM5 promoters (robust CAT clusters and robust DPI) are indicated as arrowheads. Expression profile visualized as quantitative histogram by FANTOM5 CAGE TSS (Transcription Start Sites) as the mean of rle (relative log expression) derived from ALL libraries (*n* = 1,829 samples), derived from EPC (CD14+ monocyte-derived Endothelial Progenitor Cells), and monocyte plus macrophage libraries, are shown. *C1QA, C1QB*, and *C1QC* TSS are highlighted by red, yellow, and blue boxes respectively.

**Figure 2:**
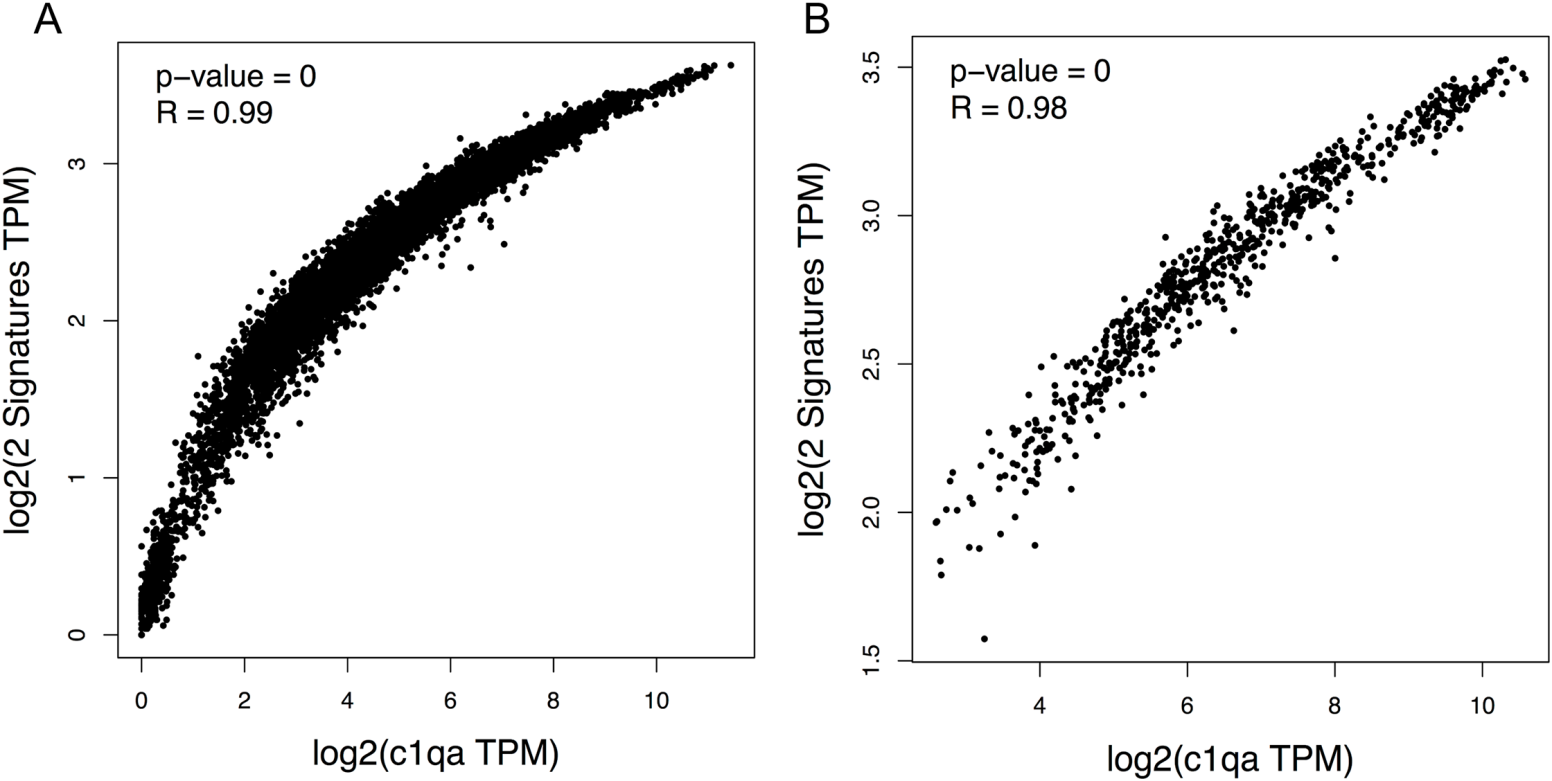
Correlation between *C1QA*, *C1QB*, and *C1QC* mRNA expression in normal tissue. Spearman correlation analysis of mRNA expression of *C1QA* vs *C1QB* and *C1QC* considered together was conducted using the GEPIA2 tool utilizing two distinct datasets, the GTEX data **(A)** or the TCGA data **(B)** of normal tissue from human samples. *C1QA* vs *C1QB* and *C1QC* were positively correlated in both dataset (P=0; R=0.99, A; P=0; R=0.99, B).

To investigate the human *C1Q* gene locus, we first used hCAGE expression data generated by the FANTOM5 Consortium. The FANTOM5 project profiles 1829 samples of a diverse range of cell types, including primary cells, cell lines, and tissues. It accurately maps promoter regions and epigenomic marks, and describes the transcriptional profile of protein-coding and long non-coding genes (38, 39). The Zenbu browser genomic data visualization tool from FANTOM-CAT (40, 41) provided us the transcriptional landscape and the dynamic expression profiles of human *C1Q* genes. Human C1q is encoded by three genes (*i.e.*, *C1QA*, *C1QB*, and *C1QC*), which are close to one another (in A-C-B order) in sense orientation on chromosome 1, as shown in **Figure 1**. As natural antisense transcription is increasingly recognized as a relevant layer of gene expression regulation (42), we searched for antisense transcription within the *C1Q* gene locus. Since we did not find any transcript signal, we could exclude the presence of C1q gene expression regulation based on natural antisense transcripts. The proximity of *C1QA*, *C1QB*, and *C1QC* genomic location underscores a potential interplay in their expression regulation. Indeed, our analysis of TSS usage across various samples revealed a consistent and significant co-expression of the three genes (**Figure 1**; **Supplementary Table 1**). Utilizing extensively FANTOM-CAT data visualization in Zenbu, we identified a robust association of *C1QA*, *C1QB*, and *C1QC* gene expression with CD14^+^ endothelial progenitor cells (EPCs), monocytes, and macrophages (**Figure 1**; **Table 1**). Functional annotations unveiled a pronounced enrichment in the immune system ontology, specifically associated with hematopoietic cells, monocytes, and macrophages (**Figure 1**; **Table 1**), emphasizing their vital role in immune response regulation. Exploring the dynamic expression patterns, all three genes exhibited significant associations with the same sample series (*i.e.*, aortic smooth muscle cell response to Fibroblast growth factor 2, macrophages response to influenza virus infection, and preadipocyte maturation). However, only *C1QB* expression showed an association for “Monocyte response upon treatment”. (**Table 2**). Moreover, we interrogated GTEx and TCGA dataset using GEPIA tool to analyze the correlation of *C1QA*, *C1QB*, and *C1QC* expression in normal samples. In both dataset, we observed a strong correlation between the expression of the three genes (**Figures 2A, B**).

**Table 1:** Sample association of *C1QA*, *C1QB*, and *C1QC* genes.

Ontology name	*C1QA*	*C1QB*	*C1QC*
Adult endothelial progenitor cells	*x*	*x*	*x*
Hematopoietic system	*x*	*x*	*x*
Hematopoietic cell	*x*	*x*	*x*
Hemolymphoid system	*x*	*x*	*x*
Hemopoietic organ	*x*	*x*	*x*
Immature conventional dendritic cell			*x*
Immune system	*x*	*x*	*x*
Leukocyte	*x*	*x*	*x*
Monocyte	*x*	*x*	*x*
Macrophage	*x*		*x*
Myeloid cell	*x*	*x*	*x*
Myeloid leukocyte	*x*	*x*	*x*
Phagocyte	*x*		
Spleen	*x*	*x*	*x*
Viscus		*x*	*x*

*x* indicates the presence of sample association with the gene expression.

**Table 2:** Dynamic expression association of *C1QA*, *C1QB*, and *C1QC* genes, (FDR, False Discovery Rate).

	Title	Series Name	Max Fold Change	FDR
***C1QA***	C1qA in AoSMC proliferation	Aortic smooth muscle cell response to FGF2	-6.38	3.08e-3
	C1qA in viral infection	Macrophage response to influenza infection	-2.25	4.45e-4
	C1qA in adipogenesis	Preadipocyte maturation	6.12	9.43e-5
***C1QB***	C1qB in AoSMC proliferation	Aortic smooth muscle cell response to FGF2	-6.37	3.12e-3
	C1qB in viral infection	Macrophage response to influenza infection	-3.28	3.4e-3
	C1qB in immune cell response -monocyte activation-	Monocyte response upon treatment	2.4	1.62e-6
	C1qB in adipogenesis	Preadipocyte maturation	7.63	1.78e-7
***C1QC***	C1qC in AoSMC proliferation	Aortic smooth muscle cell response to FGF2	-6.75	1.08e-3
	C1qC in viral infection	Macrophage response to influenza infection	-3.55	2.83e-4
	C1qC in adipogenesis	Preadipocyte maturation	7.35	1.81e-5

Max Fold Change is the maximum fold change of all comparisons within a series. AoSMC, Aortic smooth muscle cell; FGF2, Fibroblast Growth Factor 2.

### Epigenetic regulation of *C1QA*, *C1QB*, and *C1QC* gene expression across cell types

**Figure 3:**
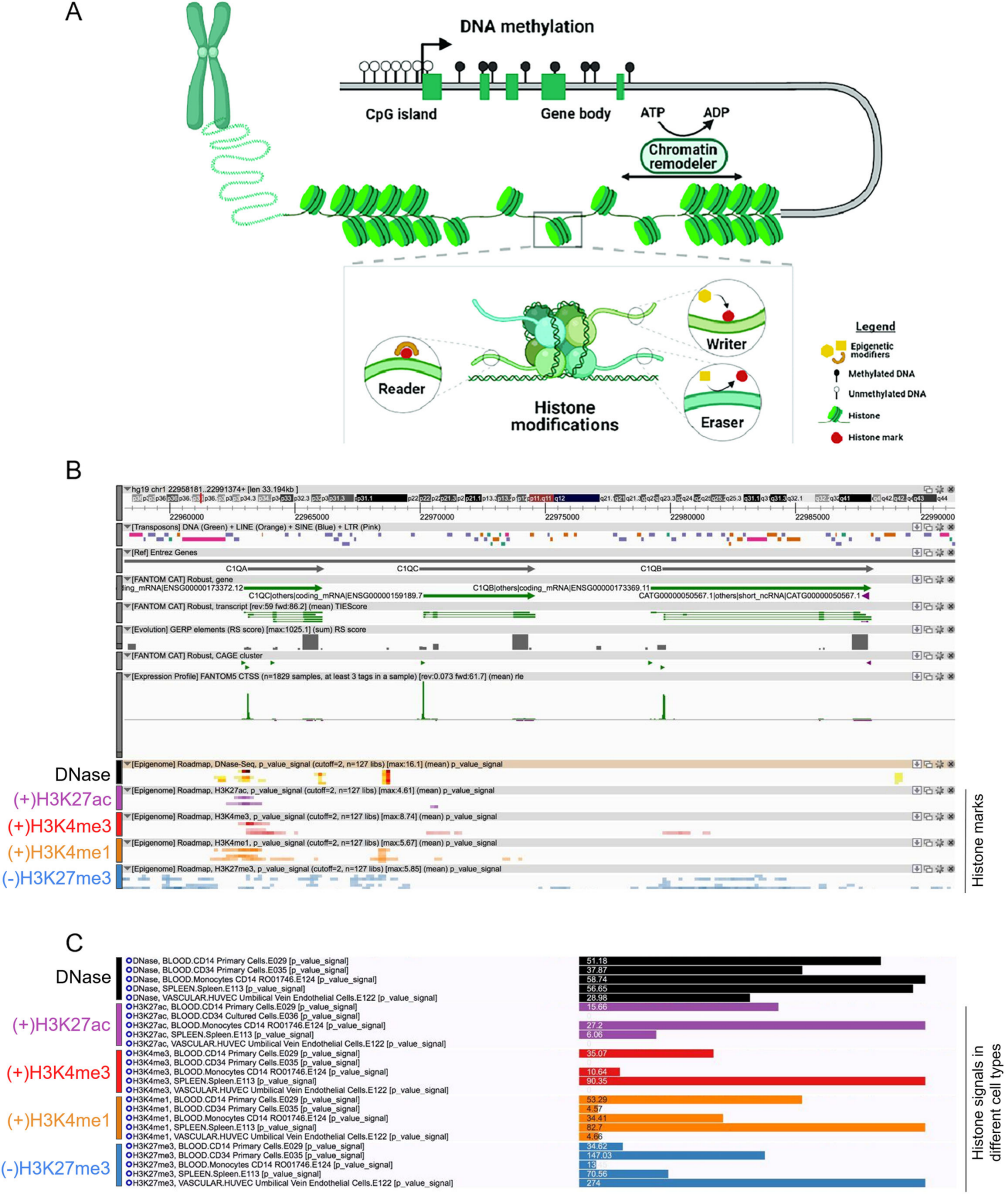
**(A)** Graphical abstract of schematic of the mechanisms of epigenetic regulation: DNA methylation and histone modification. **(A)** DNA methylation involves adding methyl groups to cytosine bases, typically at CpG sites. High levels of DNA methylation in gene promoters are usually associated with gene silencing by blocking accessibility to DNA or compacting chromatin. Histone modifications, like acetylation and methylation, affect chromatin structure. They can be added or removed by specific enzymes in response to cellular signaling, allowing for flexible and reversible regulation of gene expression **(B, C)** Fantom CAT epigenome analysis of *C1Q* gene cluster. Differential histone Chip-seq marks **(B)** and signals **(C)** was shown across different cell types on *C1Q* gene cluster from Fantom CAT. Data of DNAse I accessibility (open chromatin), H3K27ac, H3K3me3, and H3K4me1 (gene activation), and H3K27me3 (gene repression), were retrieved on *C1Q* genes.

Epigenetic modifications, including DNA methylation and histone modifications exert a crucial role in regulating gene expression without altering DNA sequence itself. DNA methylation involve adding methyl groups to cytosine bases, typically at CpG sites. High levels of DNA methylation in gene promoters are usually associated with gene silencing by blocking accessibility to DNA or compacting chromatin. Histone modifications, like acetylation and methylation, affect chromatin structure. They can be added or removed by specific enzymes in response to cellular signaling, allowing for flexible and reversible regulation of gene expression (**Figure 3A**). Moreover, they can coordinate the expression of gene clusters that should be expressed simultaneously (43). The dynamic interplay between histone modifications and activating marks plays an important role in directing cell lineage determination towards distinct functional cell types (44). Thus, we performed a comprehensive bioinformatic analysis focused on unraveling potential epigenetic regulatory mechanisms governing the expression of *C1QA*, *C1QB*, and *C1QC* genes. We utilized the online database FANTOM-CAT Epigenome to explore the dynamics of DNA accessibility and histone modifications across various cell types. Thus, we examined the promoter region of *C1QA*, *C1QB*, and *C1QC* for key histone marks indicative of open chromatin and gene activation (H3K27ac, H3K4me3, and H3K4me1), and for the repressive mark H3K27me3 as a proxy for heterochromatin and gene silencing. Concurrently, DNase-seq analysis provided insights into chromatin accessibility (**Figure 3B**). Focusing on five cell types (*i.e.*, blood CD14^+^ primary cells, blood CD34^+^ primary hematopoietic stem cells, blood monocyte CD14^+^, spleen, and HUVEC cells), we observed a distinct pattern of histone modifications (**Figure 3C**). HUVEC cells were included as a negative control for C1q synthesis (45, 46). Remarkably, blood CD14^+^ primary cells, blood monocyte CD14^+^, and spleen samples exhibited detectable levels of the histone marks associated to gene activation at the promoters of the three genes, while the repressive mark H3K27me3 displayed low levels, suggesting a permissive chromatin state in these cell types. Conversely, H3K27ac, H3K4me3, and H3K4me1 signals were notably absent or minimal in both CD34^+^ primary and HUVEC cells. This pattern coincided with elevated levels of the repressive mark H3K27me3, consistent with the non-expression of *C1QA*, *C1QB*, and *C1QC* in these cell types (**Figure 3C**). The DNase-seq data further supported these findings, revealing higher DNase activity in blood CD14^+^ primary cells, blood monocyte CD14^+^, and spleen.

### Distinct epigenetic profiles of *C1QA*, *C1QB*, and *C1QC* genes in macrophages

**Figure 4:**
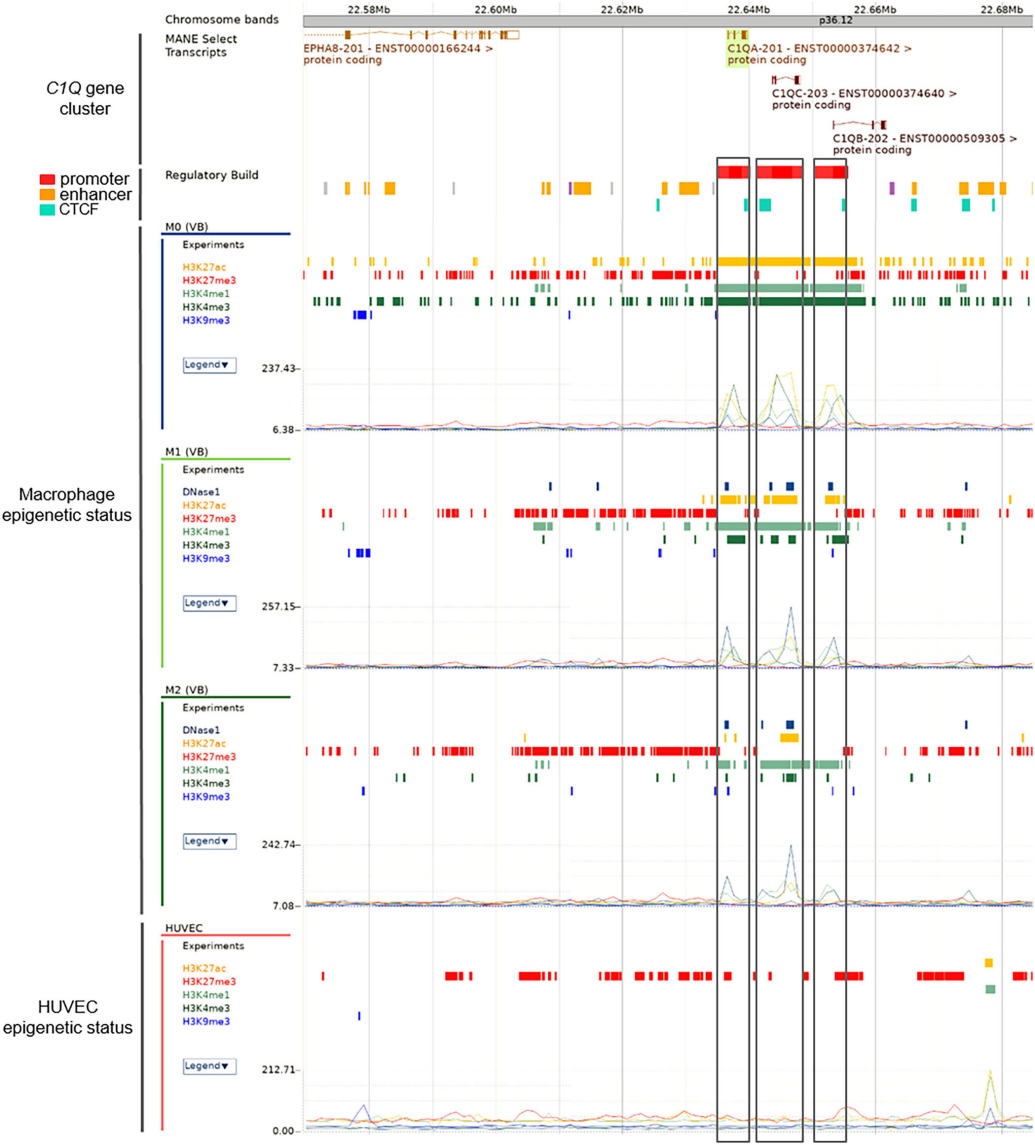
Ensembl histone mark analysis of *C1Q* gene cluster in macrophages. Differential histone Chip-seq marks on *C1Q* gene cluster was shown across M0, M1, and M2 macrophages, knowing to express *C1Q*, from Ensembl data. Promoter regions for each gene are presented as red bars and highlighted using boxes across plots of the methylation levels of H3K27ac, H2K27me3, H3K4me1, H3K4me3, and H3K9me3. HUVEC are shown as control of no *C1Q* genes expression. The signals from histone marks of transcriptional activation are present in macrophages but absent in HUVEC cells in accordance known *C1Q* genes expression in these cells.

In order to fully investigate the potential epigenetic regulation of *C1QA*, *C1QB*, and *C1QC*, we extended our analysis to incorporate data from Ensembl. a comprehensive genome browser, which offers high quality genomic analysis by integrating gene annotation, genome alignment, variants, and regulatory features (47). Specifically, we investigated the epigenetic status of macrophages, a well-known cell type for expressing *C1Q* genes. Our analysis encompasses DNAse I activity data, where available, as well as a broader spectrum of histone modifications, including H3K27ac, H2K27me3, H3K4me1, H3K4me3 (previously utilized above), and additionally, H3K9me3, a histone mark associated with gene silencing. Macrophages exhibited a distinctive epigenetic profile, characterized by a prominent presence of H3K27ac, H3K4me1, and H3K4me3, indicative of gene activation (**Figure 4**). This aligns seamlessly with known *C1QA*, *C1QB*, and *C1QC* expression patterns in macrophages. In addition, we scrutinized HUVEC cells, known for their low or absent expression of these genes (negative control). Consistent with their expression status, HUVEC cells exhibited a lack of activation histone marks for *C1QA*, *C1QB*, and *C1QC* (**Figure 4**). The inclusion of histone marks for gene silencing (*i.e.*, H3K27me3 and H3K9me3) did not provide additional discriminatory information in the epigenetic regulation of *C1QA*, *C1QB*, and *C1QC* within macrophages and HUVEC cells. While the silencing patterns remained low and consistent across the samples, the unique activation signature in macrophages suggests a distinctive epigenetic landscape.

### DNA methylation profiles of *C1QA*, *C1QB*, and *C1QC* in macrophages

**Figure 5:**
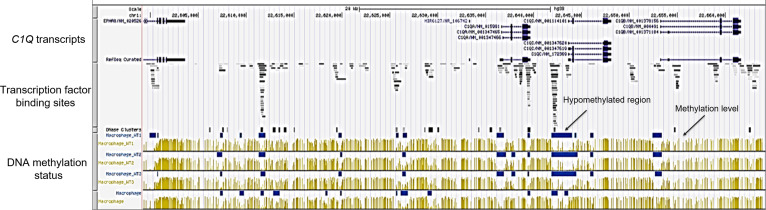
DNA methylation analysis of *C1Q* gene cluster. DNA methylation evaluation across macrophage samples on *C1Q* gene cluster was done using the methylome database MethBase on UCSC genome browser created from public BS-seq datasets. From top to bottom: chromosomal localization, *C1QA*, *C1QB*, and *C1QC* transcripts, localization of transcription factor binding site density, DNase clusters, methylation level at individual sites (in yellow) and hypomethylated regions (blues bars) for each of the four macrophage samples are shown. Hypomethylated regions are located in the promoter regions of *C1QA*, *C1QB*, and *C1QC* genes and overlap with transcription factor binding sites.

We also focused on assessing the DNA methylation levels of *C1QA*, *C1QB*, and *C1QC* using the UCSC-MethBase resource. Leveraging UCSC-MethBase, an extensive repository of DNA methylation data derived from next-generation whole-genome bisulfite sequencing, we analyzed the methylation status within the promoter regions of *C1QA*, *C1QB*, and *C1QC* in macrophages. It revealed pronounced hypomethylation across *C1QA*, *C1QB*, and *C1QC* promoter regions in macrophages (**Figure 5**). This observation aligns with the previously identified activation histone marks, suggesting a permissive chromatin state conducive to gene expression. Remarkably, the hypomethylated regions correspond with binding sites for diverse transcription factors, extending even upstream of the promoter region (**Figure 5**). This suggests a potential regulatory role for DNA methylation in the transcriptional control of these genes, introducing an additional layer of complexity to the epigenetic orchestration of *C1QA*, *C1QB*, and *C1QC* synchronized expression and their regulation.

### Epigenetic and transcriptional dynamics of *C1QA*, *C1QB*, and *C1QC* in tumors

**Figure 6:**
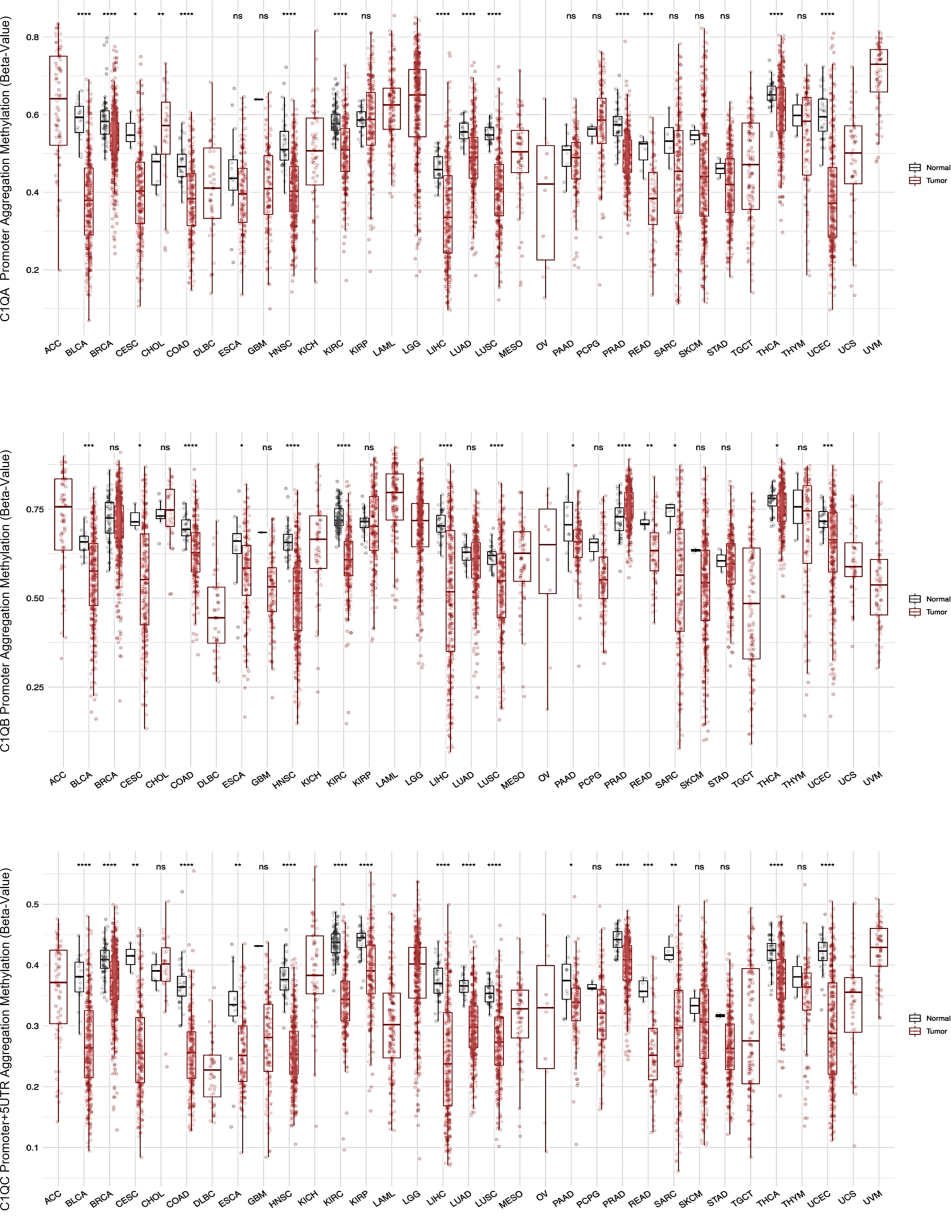
SMART analysis DNA methylation levels of *C1Q* gene cluster across different tumors and normal samples. Each of the three panel show respectively *C1QA*, *C1QB*, and *C1QC* CpG aggregated methylation across different types of tumors, evaluating the differential DNA methylation between paired tumor and normal samples, when both samples are present in the dataset. The data are presented as box plot of methylation level expressed as beta-value, in tumor sample in red and in normal sample in grey. The plots and p-values were produced using SMART tool. The sample name abbreviations are described in **Table 4**. ns, not significant. *p<0.05, **p<0.01, ***p<0.001 and ****p<0.0001.

**Figure 7:**
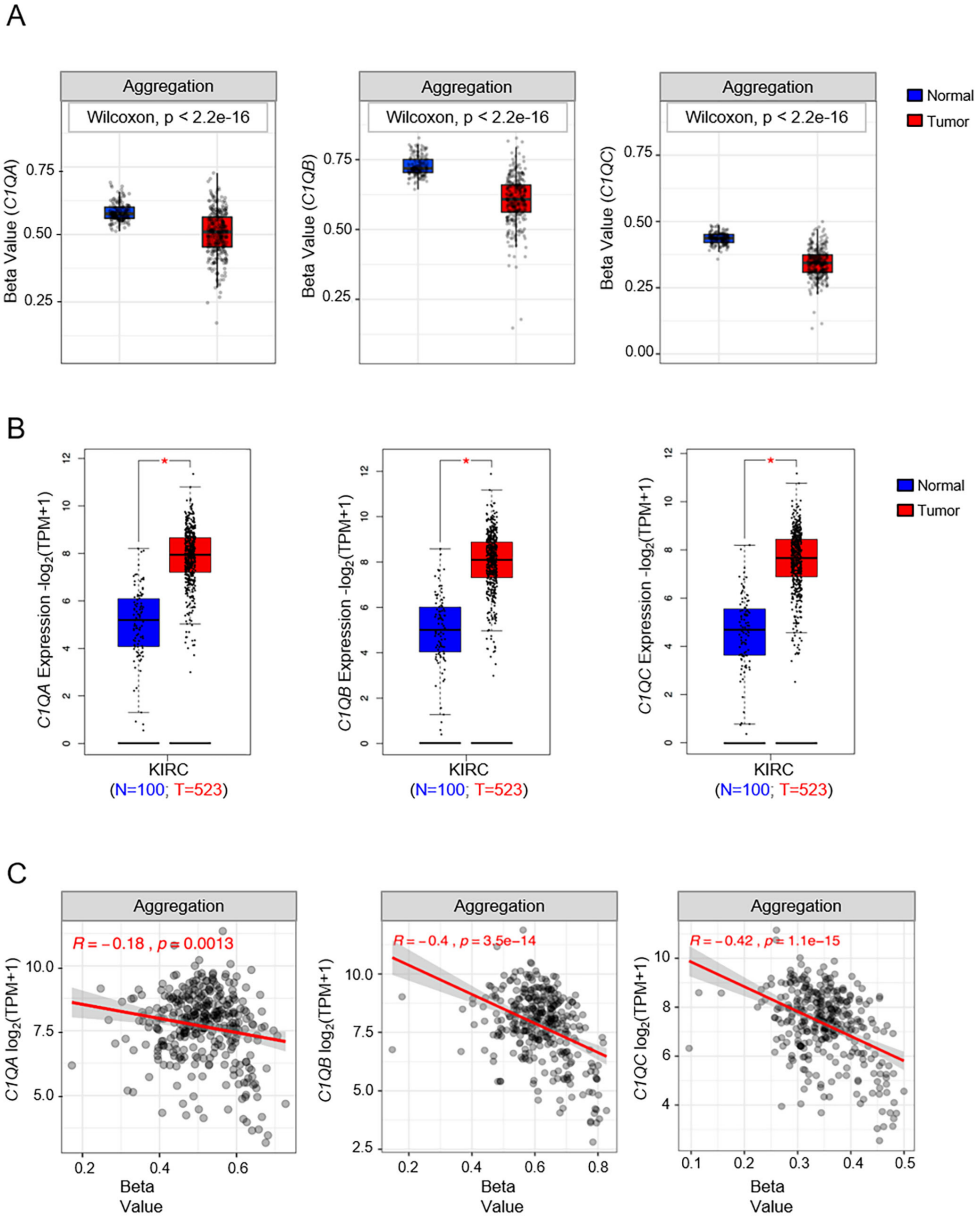
Analysis of DNA methylation and gene expression levels of *C1QA*, *C1QB*, and *C1QC* in KIRC (Kidney renal clear cell carcinoma). **(A)** SMART analysis of differentially DNA methylation levels expressed as beta-value in KIRC tumor (red box) and normal samples (blue box) for each of the *C1Q* genes. Number of tumor and normal samples are 313 and 157 respectively. **(B)** Differential gene expression analysis of *C1QA*, *C1QB*, and *C1QC* in KIRC tumor (red box) and normal samples (blue box) using GEPIA2. Number of samples used in the analysis are shown in the bottom on the plots. **(C)** SMART analysis of correlation between gene expression and DNA methylation levels expressed as beta-value in all KIRC samples (N=333). p-value and R (Pearson correlation coefficient) were calculated by tools used for the analysis. * p-value<0.01.

Our investigation into the epigenetic profiles of *C1QA*, *C1QB*, and *C1QC* by UCSC-MethBase revealed compelling insights. Remarkably, regarding tumor samples, we observed a distinct pattern of DNA methylation in tumor compared to normal samples, while analyzing colon cancer samples. The data showed higher hypomethylation in colon tumor samples compared to their normal counterparts (**Supplementary Figure 1**). This observation is consistent with earlier evidence of elevated C1q expression levels in tumors (25, 48). Employing SMART App (Shiny Methylation Analysis Resource Tool), we conducted a systematic analysis of the DNA methylation levels, represented as beta-values, within the promoters of *C1QA*, *C1QB*, and *C1QC* across 34 tumor types, including 23 with available tumor and normal samples (**Figure 6**). Notably, our analysis revealed lower methylation levels in 10 tumors compared to normal samples across all three genes, extending our previous observations in colon cancer to other tumor types (**Table 3**). To elucidate the relationship between DNA methylation status and transcriptional activation, we explored the correlation between gene expression levels and DNA methylation status for *C1QA*, *C1QB*, and *C1QC*. Strikingly, a stronger correlation was observed for *C1QB* (17/33 samples) and *C1QC* (13/33), with a comparatively lower correlation for *C1QA* (7/33) (**Table 4**). Interestingly, the samples (n = 7) where *C1QA* expression correlated with methylation levels also exhibited concordant correlations for *C1QB* and *C1QC*. As an illustrative example, we focused on Kidney Renal Clear Cell Carcinoma (KIRC), a tumor setting where we previously delineated a pro-tumorigenic role for C1q (30). Our analysis unveiled lower methylation levels of *C1QA*, *C1QB*, and *C1QC* in tumor compared to normal samples (**Figure 7A**). Leveraging GEPIA2 for gene expression data extraction, we found elevated expression of all three genes in tumor *versus* normal samples (**Figure 7B**). Intriguingly, a negative correlation was consistently observed between the expression levels of *C1QA*, *C1QB*, and *C1QC*, and their methylation status (**Figure 7C**), suggesting that these genes could become actively transcribed as methylation levels decrease and chromatin opens up.

**Table 3:** Summary of differential DNA methylation levels in tumor and normal samples analyzed with SMART tool.

Abbreviation	Study Name	n. tumor samples	n. normal samples	*C1QA* MethDiff	*C1QB* MethDiff	*C1QC* MethDiff
**BLCA**	**Bladder Urothelial Carcinoma**	**413**	**21**	****	***	****
**BRCA**	**Breast invasive carcinoma**	**783**	**87**	****	**ns**	****
**CESC**	**Cervical squamous cell carcinoma and endocervical adenocarcinoma**	**308**	**3**	*	*	**
**CHOL**	**Cholangiocarcinoma**	**36**	**9**	** (^)	**ns**	**ns**
**COAD**	**Colon adenocarcinoma**	**288**	**34**	****	****	****
**ESCA**	**Esophageal carcinoma**	**184**	**15**	**ns**	*	**
**GBM**	**Glioblastoma multiforme**	**150**	**1**	**ns**	**ns**	**ns**
**HNSC**	**Head and Neck squamous cell carcinoma**	**525**	**50**	****	****	****
**KIRC**	**Kidney renal clear cell carcinoma**	**313**	**157**	****	****	****
**KIRP**	**Kidney renal papillary cell carcinoma**	**272**	**43**	**ns**	**ns**	****
**LIHC**	**Liver hepatocellular carcinoma**	**376**	**50**	****	****	****
**LUAD**	**Lung adenocarcinoma**	**458**	**30**	****	**ns**	****
**LUSC**	**Lung squamous cell carcinoma**	**364**	**41**	****	****	****
**PAAD**	**Pancreatic adenocarcinoma**	**184**	**10**	**ns**	*	*
**PCPG**	**Pheochromocytoma and Paraganglioma**	**183**	**3**	**ns**	**ns**	**ns**
**PRAD**	**Prostate adenocarcinoma**	**496**	**50**	****	**** (^)	****
**READ**	**Rectum adenocarcinoma**	**93**	**7**	***	****	***
**SARC**	**Sarcoma**	**261**	**4**	**ns**	*	**
**SKCM**	**Skin Cutaneous Melanoma**	**473**	**2**	**ns**	**ns**	**ns**
**STAD**	**Stomach adenocarcinoma**	**393**	**2**	**ns**	**ns**	**ns**
**THCA**	**Thyroid carcinoma**	**511**	**53**	****	*	****
**THYM**	**Thymoma**	**124**	**2**	**ns**	**ns**	**ns**
**UCEC**	**Uterine Corpus Endometrial Carcinoma**	**419**	**33**	****	***	****

Consistently, DNA methylation levels are significantly lower in tumor samples with respect to normal samples unless otherwise indicated (^ = DNA methylation levels are significantly lower in normal samples with respect to tumor samples). The p-value (MethDiff) were produced using SMART analysis, ns, not significant, *p<0.05, **p<0.01, ***p<0.001 and ****p<0.0001. Highlighted in yellow are samples that show statistically significant lower DNA methylation in tumor with respect to normal samples for each of the three genes.

**Table 4:** Correlation analysis of DNA methylation and gene expression levels in different samples using SMART tool.

Expression/Methylation Correlation				
Abbreviation	Study name	*C1QA*	*C1QB*	*C1QC*
ACC	Adrenocortical carcinoma	negative	negative	negative
BLCA	Bladder Urothelial Carcinoma	positive	no	no
BRCA	Breast invasive carcinoma	no	negative	negative
CESC	Cervical squamous cell carcinoma and endocervical adenocarcinoma	no	negative	no
CHOL	Cholangiocarcinoma	positive	no	no
COAD	Colon adenocarcinoma	positive	no	no
DLBC	Diffuse Large B-cell Lymphoma	no	no	no
ESCA	Esophageal carcinoma	positive	no	no
GBM	Glioblastoma multiforme	no	no	no
HNSC	Head and Neck squamous cell carcinoma	positive	positive	positive
KICH	Kidney Chromophobe	no	negative	negative
KIRC	Kidney renal clear cell carcinoma	negative	negative	negative
KIRP	Kidney renal papillary cell carcinoma	negative	negative	negative
LAML	Acute Myeloid Leukemia	no	negative	no
LGG	Brain Lower Grade Glioma	negative	negative	negative
LIHC	Liver hepatocellular carcinoma	positive	no	no
LUAD	Lung adenocarcinoma	positive	negative	no
LUSC	Lung squamous cell carcinoma	positive	no	positive
MESO	Mesothelioma	no	negative	negative
OV	Ovarian serous cystadenocarcinoma	no	no	no
PAAD	Pancreatic adenocarcinoma	positive	negative	no
PCPG	Pheochromocytoma and Paraganglioma	no	no	no
PRAD	Prostate adenocarcinoma	positive	negative	negative
READ	Rectum adenocarcinoma	positive	no	positive
SARC	Sarcoma	no	negative	negative
SKCM	Skin Cutaneous Melanoma	no	no	no
STAD	Stomach adenocarcinoma	positive	no	positive
TGCT	Testicular Germ Cell Tumors	no	negative	negative
THCA	Thyroid carcinoma	negative	negative	negative
THYM	Thymoma	negative	negative	negative
UCEC	Uterine Corpus Endometrial Carcinoma	positive	no	no
UCS	Uterine Carcinosarcoma	no	no	no
UVM	Uveal Melanoma	negative	negative	negative

Samples with negative correlation for all the three *C1Q* genes are highlighted in pink. Samples with at least one of the three *C1Q* genes showing negative correlation are highlighted in blue.

## Discussion

The complement system plays a critical role in the immune response, bridging innate and adaptive immunity (1, 2). Among its components, C1q stands out as a key versatile molecule, initiating the classical pathway and participating in processes such as efferocytosis, differentiation, chemotaxis, aggregation, and adhesion (49). Understanding the regulation of C1q expression is crucial for elucidating its ambivalent roles in health and disease. Its expression at the tissue level plays an important role in physiological processes, such as trophoblast invasion during the first trimester of pregnancy (22) and synaptic pruning (50); at the same time, it plays pivotal roles in pathological processes, such as wound healing (23), tumor development (25, 48), autoimmune diseases, and endometriosis (51). Few studies on the transcriptional regulation of *C1Q* have been published: they identified specific transcription factors that can activate *C1Q* promoters such as PU.1. PU.1, an Ets-family transcription factor, is required for the development of hematopoietic myeloid lineage immune cells (52). The transcription factors PU.1 and IRF8 bind to a 53-bp element on *C1QB* promoter known to be a gene expression regulatory region (27). PU.1 influences *C1Q* expression in the decidua during placental development, and its presence is directly correlated with C1q synthesis (28). Additionally, the transcription factor, MafB, specifically expressed in monocytes and macrophages, binds to and activates the transcription of all three *C1Q* chain genes. *C1Q* is expressed at the microenvironment level in different types of gliomas with an ambivalent prognostic significance (30, 31, 53). Its crucial role has been widely demonstrated in mesothelioma, where in addition to directly promoting the proliferation and resistance of tumor cells (48), it modulates the metabolism of hyaluronic acid (54, 55). However, our understanding of how *C1Q* expression is modulated, particularly in the tumor microenvironment, remains limited.

Previous evidence revealed a synchronized transcription of *C1Q* genes (27). Our integrated analysis of TSS usage and expression patterns of the human *C1Q* genes (*C1QA*, *C1QB*, and *C1QC*) via Zenbu browser and FANTOM-CAT data highlighted a coordinated regulation and functional association across various cell types. This co-expression pattern, particularly prominent in immune-related cells (*e.g.*, monocytes and macrophages), underscores the importance of C1q in immune regulation. Moreover, the functional annotation-based analysis confirmed C1q as one of the few non-liver-derived complement components whose major expression can be traced back to the bone marrow (56). Its expression in macrophages is also exemplified by recent identification of C1q+ macrophage populations in both healthy and tumor tissues (57). The evidence that, in certain cell populations, the expression of only one or two chains of C1q has been observed may not have a functional significance, but could be a spurious expression linked to the ancestral role of C1q as an acute phase molecule (24, 45).

Of interest is the association between *C1QA*, *C1QB*, and *C1QC* gene expression and CD14^+^ EPCs. We previously demonstrated the role of C1q in angiogenesis (21, 23), and its presence in several microenvironments characterized by active angiogenesis, such as decidua, endometriotic lesions, and tumors (22, 25, 51). These novel findings lead us to hypothesize that CD14^+^ EPCs could be the primary cells responsible for C1q synthesis in angiogenic regions.

Epigenetic modifications, including histone modifications and DNA methylation, play a central role in determining gene expression patterns. First, the analysis of histone modification patterns across different cell types revealed distinct epigenetic landscapes associated with *C1Q* expression. Specifically, permissive chromatin states characterized by activation histone marks are observed in cell types where *C1Q* is expressed (*i.e.*, macrophages), while repressive marks dominate in non *C1Q*-expressing cell types (*i.e.*, HUVEC cells). These findings suggest a direct link between chromatin state and transcriptional activity of *C1Q* genes. Moreover, examining DNA methylation profiles further elucidates the epigenetic regulation of *C1Q* expression. In fact, hypomethylation of the promoter regions in macrophages correlates with active transcription. The macrophage-specific activation signature further emphasizes the correlation between epigenetic marks and gene expression. In support of this evidence, the regulatory roles of histone and DNA modifications have recently emerged as crucial contributors to the monocyte and macrophage functions (58–60). The transition from monocyte to macrophage is marked by a specific gene expression profile, concomitant with extensive alterations in histone modification and chromatin accessibility (61). These findings underscore the indispensable involvement of epigenetic mechanisms in endowing macrophages with the capacity for phenotypic plasticity and functional versatility (62). Of interest, an epigenetic program is associated with monocyte-to-macrophage differentiation (61).

Previous reports have extensively emphasized a regulatory interdependence between C1q and microenvironment. Thus, C1q shapes the monocyte/macrophage/DC phenotype towards the development of a pro-efferocytic and anti-inflammatory phagocyte, creating an anti-inflammatory feed-forward loop (63), but also the microenvironment can influence C1q production. Unlike the monocyte-macrophage lineage, which constitutively produces C1q and modulates its expression in response to microenvironmental stimuli [*e.g.*, the shift towards tumor-associated macrophages (TAMs)], some cells abruptly initiate or interrupt C1q production during their maturation process. Extravillous trophoblasts start expressing C1q during maturation and differentiation (24) via an active involvement of epigenetic dynamics in trophoblast biology (64). Conversely, the sustained production of active C1q by immature DCs is completely down-regulated upon DC maturation and differentiation *in vitro* (19). These findings strengthen our evidence of a fine regulation of *C1Q* expression in differentiation contexts.

We also aimed at shedding light on the ambivalent role of C1q in the tumor microenvironment. C1q has already been demonstrated to serve as a pro-tumorigenic factor in several tumor settings (25, 48, 54, 55, 65–67). In KIRC, infiltration by C1q-producing TAMs was associated with an immunosuppressed tumor microenvironment, characterized by high expression of immune-checkpoint molecules (PD-1, LAG-3, PD-L1, and PD-L2) (66, 68). Nevertheless, the prognostic impact of C1q is ambivalent and strictly dependent on the tumor type (30, 31, 51).

Interestingly, we found that tumor samples exhibit a distinct DNA methylation pattern compared to normal tissues, with decreased methylation associated with elevated *C1Q* expression, in accordance with previous studies (25, 65–67). Our observations suggest a potential role for epigenetic dysregulation in *C1Q* expression during tumorigenesis, and underscore the dynamic interconnection between DNA methylation and gene expression in *C1QA*, *C1QB*, and *C1QC* across various tumor types, shedding light on the intricate relationship between epigenetic modifications and the switching of transcriptional activation and deactivation processes. These findings not only substantiate the observed epigenetic patterns in macrophages but also emphasize the broader implications of DNA methylation in the transcriptional regulation of these immune response genes.

## Conclusions

Overall, the comprehensive analysis of *C1Q* gene regulation sheds light on the intricate interplay between epigenetic mechanisms and transcriptional dynamics in modulating immune responses and disease processes. Further investigation into the epigenetic regulation of *C1Q* expression may offer new insights into the pathogenesis of immune-related disorders and cancer, providing potential therapeutic targets for intervention.

## Data Availability

The original contributions presented in the study are included in the article/**Supplementary Material**. Further inquiries can be directed to the corresponding author.
