# Supplementary material for: Epigenetic regulation of complement C1Q gene expression
Source: Front Immunol. 2024 Dec 3;15:1498097. doi: 10.3389/fimmu.2024.1498097 (PMC11653359; doi:10.3389/fimmu.2024.1498097)
Supplement: Supplementary file 1 [file DataSheet1.docx]

Supplementary Material to

**Epigenetic regulation of complement C1q gene expression**

Silvia Pegoraro^1^, Andrea Balduit^1^, Alessandro Mangogna^1^, Uday Kishore^2,3^, Giuseppe Ricci^1,4^, Chiara Agostinis^1^ and Roberta Bulla^5^

^1^ Institute for Maternal and Child Health, IRCCS Burlo Garofolo, Trieste, Italy

^2^ Department of Veterinary Medicine, U.A.E. University, Al Ain, U.A.E.

^3^ Zayed Centre for Health Sciences, United Arab Emirates University, Al Ain, U.A.E.

^4^ Department of Medical, Surgical and Health Science, University of Trieste, Trieste, Italy

^5^ Department of Life Sciences, University of Trieste, Trieste, Italy

**Corresponding author:**

**Running Title:** Epigenetic regulation of C1q.

**Key words:** epigenetic regulation, C1q, *in silico* analysis, *C1QA, C1QB, C1QC.*

**Supplementary Table 1.** Summary of the sample and dynamic expression association of *C1QA*, *C1QB*, and *C1QC* genes, indicating a huge overlap.

| **Gene** | **Sample Ontology Association** | **Association in Dynamic Expression** |
| --- | --- | --- |
| ***C1QA*** | Yes (13) Best: EPC | Yes (3) |
| ***C1QB*** | Yes (12) Best: EPC | Yes (4) |
| ***C1QC*** | Yes (14) Best: EPC | Yes (3) |
| ***C1Q A/B/C*** | 11 in common | 3 in common |

**
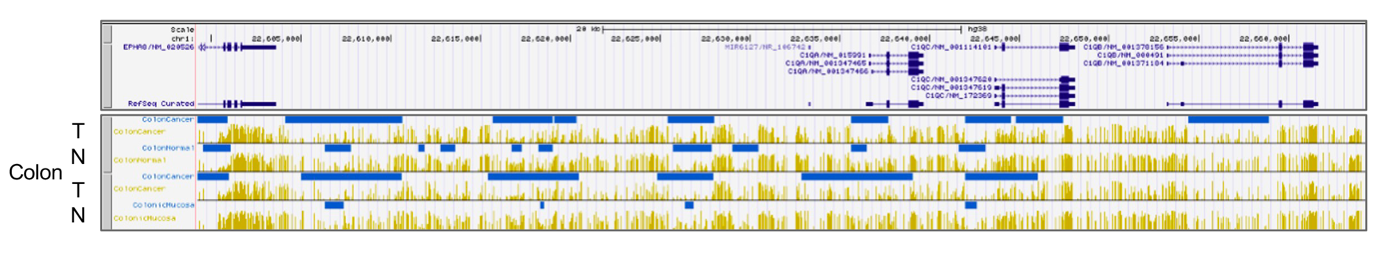
**

**Supplementary Figure 1. DNA methylation analysis of C1q gene cluster in colon cancer.** Differential DNA methylation levels between tumor and normal colon samples is shown using the methylome database MethBase on UCSC genome browser created from public BS-seq datasets. Methylation level at individual sites (in yellow) and hypomethylated regions (blues bars) are shown.
